# Rationale and Design of a Panel Study Investigating Six Health Effects of Airborne Pollen: The EPOCHAL Study

**DOI:** 10.3389/fpubh.2021.689248

**Published:** 2021-06-18

**Authors:** Alexandra Bürgler, Sarah Glick, Karin Hartmann, Marloes Eeftens

**Affiliations:** ^1^Swiss Tropical and Public Health Institute, Basel, Switzerland; ^2^University of Basel, Basel, Switzerland; ^3^Division of Allergy, Department of Dermatology, University Hospital Basel, University of Basel, Basel, Switzerland; ^4^Department of Biomedicine, University Hospital Basel, University of Basel, Basel, Switzerland

**Keywords:** airborne pollen, climate change, allergic rhinitis, cardiovascular health, pulmonary health, cognitive performance, health-related quality of life, sleep

## Abstract

**Background:** While airborne pollen is widely recognized as a seasonal cause of sneezing and itchy eyes, its effects on pulmonary function, cardiovascular health, sleep quality, and cognitive performance are less well-established. It is likely that the public health impact of pollen may increase in the future due to a higher population prevalence of pollen sensitization as well as earlier, longer, and more intense pollen seasons, trends attributed to climate change. The effects of pollen on health outcomes have previously been studied through cross-sectional design or at two time points, namely preceding and within the period of pollen exposure. We are not aware of any observational study in adults that has analyzed the dose-response relationship between daily ambient pollen concentration and cardiovascular, pulmonary, cognitive, sleep, or quality of life outcomes. Many studies have relied on self-reported pollen allergy status rather than objectively confirming pollen sensitization. In addition, many studies lacked statistical power due to small sample sizes or were highly restrictive with their inclusion criteria, making the findings less transferable to the “real world.”

**Methods:** The EPOCHAL study is an observational panel study which aims to relate ambient pollen concentration to six specific health domains: (1) pulmonary function and inflammation; (2) cardiovascular outcomes (blood pressure and heart rate variability); (3) cognitive performance; (4) sleep; (5) health-related quality of life (HRQoL); and (6) allergic rhinitis symptom severity. Our goal is to enroll 400 individuals with diverse allergen sensitization profiles. The six health domains will be assessed while ambient exposure to pollen of different plants naturally varies. Health data will be collected through six home nurse visits (at approximately weekly intervals) as well as 10 days of independent tracking of blood pressure, sleep, cognitive performance, HRQoL, and symptom severity by participants. Through repeated health assessments, we aim to uncover and characterize dose-response relationships between exposure to different species of pollen and numerous acute health effects, considering (non-)linearity, thresholds, plateaus and slopes.

**Conclusion:** A gain of knowledge in pollen-health outcome relationships is critical to inform future public health policies and will ultimately lead toward better symptom forecasts and improved personalized prevention and treatment.

## Introduction

Climate change has greatly impacted the onset, duration and intensity of the pollen season in recent decades, leading to an increase in exposure to some allergenic pollen species such as birch, hazel, oak, beech, and nettle and hemp families in Switzerland (1, 2). Similar shifts have been observed in other countries (3, 4). At the same time, allergies to airborne pollen are increasingly common in Europe (5, 6), and prevalence in Switzerland has risen from around 0.82% in 1926 to between 14 and 20%, as estimated by more recent studies (7–10).

The common nasal and ocular symptoms of pollen allergy, known as *intermittent allergic rhinitis* (IAR) or colloquially as hay fever, are easily recognized but sometimes trivialized by patients (11). IAR is an allergic inflammatory condition, where histamine release activates a cascade of inflammatory cells and molecules. While the localized effects of histamine (eye and nose itching, nasal congestion and discharge, eye redness) are well-understood, much less is known about the systemic health impacts of pollen-mediated inflammation. It is not generally recognized that high pollen concentrations may increase respiratory and cardiovascular events, leading to excess hospitalizations (12), impacts on quality of life (13), productivity loss (14), poorer school performance (15) and economic expenses. In the European Union (EU), economic costs of allergy-related work absence and reduced working capacity amount to €55–151 billion per year (16). Consequently, it is important to assess the wide-ranging health effects of pollen, which will likely become even more prominent in the future due to climate change.

Firstly, this manuscript aims to give an overview of epidemiological research conducted on pollen and six specific health outcomes: (1) pulmonary function and inflammation; (2) cardiovascular outcomes (blood pressure and heart rate variability); (3) cognitive performance; (4) sleep; (5) health-related quality of life (HRQoL); and (6) allergic rhinitis symptom severity. We highlight limitations of previous studies and identify gaps in knowledge, thereby providing the rationale for the EPOCHAL study (Effects of Pollen on Cardiorespiratory Health and Allergies). Secondly, this paper describes the design of the EPOCHAL panel study, which aims to quantify and characterize how ambient pollen concentration affects the aforementioned six health domains. Dose-outcome relationships in our study population will be investigated, looking specifically at (non-)linearity, thresholds, and plateaus.

In addition, we aim to study:

1. How does sensitization to at least one plant pollen, demonstrated by positive skin prick test (SPT), affect the outcomes within the six health domains?
  - How do sensitizations to particular plants differentially affect the six health outcomes?
  - In what ways are health outcomes measurably different in pollen monosensitized vs. polysensitized individuals?
  - How is an increasing number of plant pollen sensitizations on the SPT related to the health outcomes?
2. Do individuals with higher self-reported severity of allergic rhinitis symptoms manifest variant health outcomes in the other five health domains?
3. Are there subgroups with distinct dose-response relationships between pollen exposure and health outcomes? (e.g., age, gender, asthma comorbidity, etc.)
4. Are there synergies between pollen intensity and other exposure variables (weather, air pollution) in their effect on the health outcomes?
5. Is there a measurable effect of variable pollen exposure on the six health outcomes among individuals without a pollen sensitization?

## Study Rationale for the Six health outcomes

### Pulmonary Outcomes

Individuals with allergic rhinitis (AR) frequently have co-existing asthma (estimated at 10–40%) (17), while another 45% of AR individuals without asthma have bronchial hyperresponsiveness (18), an intermediate step toward asthma development. Selected studies have observed that individuals with AR can have an abnormal pulmonary function test (PFT) (19–24) and fractional excretion of nitric oxide (FeNO) (25–29) even in the absence of asthma, suggesting that pollen is an important trigger for lower airway inflammation. In addition, FeNO has been shown to increase in sensitized individuals at times of higher pollen counts (27) and has been associated with the number of positive reactions on the SPT (25). However, to our knowledge, there are no cohort studies in adults with AR which repeatedly measure pulmonary function and FeNO at various ambient levels of pollen intensity.

### Cardiovascular Outcomes

#### Blood Pressure

Two European cohort studies have noted a modest but significant elevation in systolic BP (3–6 mm Hg) in adults with AR vs. controls (30, 31). However, this effect has not been replicated in subsequent research (31–36) and hence the overall evidence remains inconsistent. Since elevated BP is a risk factor for myocardial infarction and stroke, and several epidemiological studies have found increased mortality due to cardiovascular causes on high pollen days (12, 37), any potential pollen-related effect on BP is crucially important to understand. Prior research evaluating this potential association has been limited by: BP data collection at a single encounter (30–36); not specifying whether BP was measured during or outside of pollen season (30–36); and/or reliance on self-reported AR without confirmatory allergy testing (30–35). To our knowledge, air pollution has not been considered as a confounder in any previous AR-BP studies.

#### Heart Rate Variability

Heart rate variability (HRV) describes the changeability of time intervals between two heart beats and reflects a dynamic autonomic nervous system balance that is influenced by sympathetic and parasympathetic nervous system activity (38). HRV is sensitive to acute physiological changes and is therefore a suitable and interesting measure to investigate cardiovascular health in relation to pollen exposure. A lower HRV, reflecting an increased sympathetic (39) and/or diminished parasympathetic (vagal) tone (40), has been associated with heart disease, major depressive disorder (40), and increased risk of mortality (39). An autonomic imbalance or dysfunction may also play a role in the pathophysiology of allergic rhinitis (41, 42). There is a limited number of epidemiological studies of allergic populations, which have shown mixed results (43, 44). However, these studies predominantly suggest higher parasympathetic (45–48) and lower sympathetic (43, 47–50) activity when compared to non-allergic controls. It has been proposed that decreased sympathetic activity can lead to a pro-inflammatory state in other organ systems (42), of interest to measured health outcomes beyond the upper respiratory tract. All prior AR-HRV studies are constrained by small sample sizes (20–50 participants) or measurement of HRV during a single day. Additionally, these studies do not represent a real-life AR population because most required abstaining from allergy medication for multiple weeks or excluded asthmatics and individuals with other atopic diseases, common co-occurring conditions with AR.

### Cognitive Performance

Cognition could be modulated in individuals with AR via sleep impairment, medication side effects, disrupted mood and/or the actions of pro-inflammatory cytokines (51, 52) and histamine released during allergic inflammation (53). Various cognitive domains have been compared in individuals with and without AR. There are mixed findings for whether there is a significant difference between these populations in the areas of vigilance, attention, verbal learning, and working memory (13, 53–56). However, there is stronger evidence for diminished processing speed in allergic individuals during the pollen season (10–20% decrease compared to healthy controls) (53, 55, 56). A number of studies have assessed pollen exposure effects, typically comparing within- and outside-pollen season, but they are constrained by small study populations (40–80 participants) (53, 54, 56, 57) and/or a limited number of time points analyzed (13, 53–56). Two ecological studies (15, 58) have investigated the impact of daily pollen concentrations on high school test performance and found that the average students' test scores were reduced by 0.85% when pollen level increased by one standard deviation from the mean (after log-transformation). However, a major drawback of ecological studies is that intermittent AR prevalence was estimated, and no conclusions about cognitive effects could be drawn at the individual level.

### Sleep

Three mechanisms that could contribute to the influence of AR on sleep are: direct effect of inflammatory mediators such as histamine (59) and cytokines (60), allergic rhinitis symptoms (foremost nasal congestion) (61), and autonomic system dysfunction (62, 63) (see section Heart Rate Variability). A large European survey of individuals with intermittent AR demonstrated that more than half had self-reported sleep impairment related to their symptoms, including: trouble falling asleep (52.3%); nighttime awakening (51.8%); staying asleep (50.8%); and insufficient sleep (60.3%) (64). In addition, a recent meta-analysis of observational studies found that patients with AR show no significant differences in sleep duration or sleep stages but have overall decreased subjective sleep quality and more frequent problems with insomnia, restless sleep, daytime sleepiness, and the use of sleep medication (63). However, the strength of the evidence is debatable given that AR status was often self-reported (without confirmatory pollen sensitization testing) and that conclusions for some outcomes were based on few studies (example: sleep stages). Further, the researchers relied on self-reported sleep duration and quality metrics, which are subject to recall bias and social desirability.

### Health-Related Quality of Life

The inflammatory process in AR involves cytokines, messenger molecules that can interact with the brain and cause changes in mood (65), anxiety (51), fatigue, psychomotor slowing and sleep impairment (66). HRQoL studies in populations with intermittent AR have consistently demonstrated a reduction across several domains, importantly physical, emotional, functional, and psychological health (64, 67–70). AR has been implicated in problems with social activities, decreased mood and irritability, disrupted sleep, daytime fatigue, school issues due to difficulty with learning, workplace absenteeism, decreased perceived control of health, activity restrictions, and anxiety related to increased physician visits and medication costs (14, 68–70). In a European survey from 2007, more than 80% of individuals with intermittent AR reported at least one HRQoL impairment attributable to their disease (64). While AR-related HRQoL effects appear to be independent of age or income, at least one study has suggested that females may be more affected (67). To our knowledge, there are no studies which have repeatedly assessed HRQoL outcomes and related the results to airborne pollen exposure.

### Allergic Rhinitis Symptom Severity

A number of cohort studies have investigated the relationship between pollen concentrations and allergic rhinitis symptom severity and occurrence (71–74). Mapping AR symptom intensity through app-based “citizen science” platforms has also been explored, as these metrics can correlate well with real-time, local pollen concentration (75, 76). In a meta-analysis of 12 studies, it was shown that the risk of lower respiratory, upper respiratory, ocular, asthmatic and general allergic symptoms increased between 1 and 11% when pollen exposure increased by 10 grains per cubic meter (m^3^) (74). Studies have come to different conclusions regarding the existence of a pollen threshold to evoke symptoms (71–73) but agree on a sharp increase of allergy symptoms at the beginning of pollen season (72, 77). More evidence is needed to understand the nature of such thresholds as well as the shape of the dose-response relationship between pollen concentration and AR symptoms, for different pollen species.

### Air Pollution, Weather, and Pollen Interactions

Epidemiological studies have not yet explored the interactions of anthropogenic air pollution and allergic rhinitis symptoms other than respiratory symptoms (78). For acute respiratory outcomes, the epidemiologic evidence remains unclear for allergen-pollution interactions. However, there is stronger evidence for an interaction in experimental panel studies with humans. Allergic rhinitis symptoms have been significantly associated with moderate levels of air pollutants, for example with nitrogen oxides (NO_x_) and ozone (O_3_) (71). Furthermore, NO_2_, O_3_, PM_10_ (particles) and sulfur dioxide (SO_2_) have been shown to interact with pollen in aggravating symptoms of asthma (79, 80). More heterogeneous findings have been reported by toxicological studies which studied allergen expression on pollen grains from trees growing under different environmental conditions, finding that SO_2_ inhibits allergen expression (81), whereas higher ozone concentrations lead to more allergen expression as well as higher allergenicity confirmed by SPT (82).

Beyond air pollution, weather can modulate pollen allergy symptoms. Thunderstorms with co-occurring extreme grass pollen concentrations have been associated with an escalation of asthma- and respiratory-related hospital admissions of individuals who were highly sensitized to grass pollen (e.g., Melbourne thunderstorm asthma epidemic) (83, 84). The aforementioned pulmonary, cardiovascular, cognition, sleep, and HRQoL bodies of literature have uncommonly considered how air pollution and/or weather may have modified outcomes attributed to pollen.

### Research Gaps

In summary, among the six outcomes of interest, we identified the following research gaps:

- There is a paucity of prospective observational studies which collect health outcome data at more than 2 time points;
- Previous studies largely considered environmental exposure to pollen in a dichotomous manner (“in” vs. “out” of pollen season) rather than a continuous variable, which does not allow for dose-response analyses between pollen concentration and health outcomes;
- Most studies do not consider personal pollen sensitization profile on SPT, but instead rely on self-reported pollen allergy;
- Many studies have a lack of statistical power due to their small sample sizes;
- The inclusion criteria of many studies are very restrictive, limiting the generalizability of results to a “real world” population, particularly for allergy medication users, pollen polysensitized individuals, or adults with both AR and asthma;
- Other environmental pollutants (e.g., weather, air pollution) are rarely considered as confounders;
- Little is known about the health effects of pollen on individuals without AR.

## Methods and Analysis

### Design

The EPOCHAL study is an observational and longitudinal panel study conducted in Basel, Switzerland, with two recruitment periods, from February to end of August in 2021 and the same months in 2022. The chosen months of data collection cover the most typical pollen seasons for trees, grasses, and weeds in the Basel region. We aim to include 400 participants overall. The duration of study enrolment per individual will be approximately 6 weeks.

### Selection of Subjects

This panel study will include adults who are between 18 and 65 years old and live within a 40-min commute from Basel-Stadt. The study panel will be sex-balanced and reflect the full pollen allergy spectrum, including individuals who are non-sensitized; sensitized but asymptomatic; monosensitized with symptomatic IAR; and polysensitized with symptomatic IAR. We aim to include ~300 adults with a health history of pollen-related allergic rhinitis and 100 individuals without pollen symptomatology. EPOCHAL is a real-world observational study which restricts participants minimally in their use of allergy and non-allergy medications. Nevertheless, one important exclusion to study participation is receipt of pollen immunotherapy within the previous 5 years. Study participants must also agree to short-term abstinence of oral antihistamines for 7 days prior to the SPT. These two restrictions on prior/current allergy treatment are meant to ensure the validity and reliability of data.

Adults with asthma or preexisting high blood pressure are welcome to participate. However, we will exclude individuals with major, pre-existing cardiac and pulmonary conditions as well as epilepsy. Visual or hearing loss and restricted ability to complete the cognitive tests independently (e.g., dementia) will also lead to exclusion. Furthermore, persons who are pregnant, regular users of medications which suppress the immune system (either oral, intravenous or injection, e.g. for rheumatoid arthritis, lupus, inflammatory bowel disease, or another autoimmune condition) and people who cannot refrain from psychoactive drug use for the duration of the study will not be enrolled.

Recruitment channels will include the Division of Allergy of the University Hospital Basel; newsletters of the aha! Swiss Allergy Center; advertisements in newspapers; student, Swiss TPH and general websites; and social media posts and stories (Instagram, Facebook, Twitter). Direct personal contact between the study nurses and participants from the start of recruitment and flexible, at-home health assessment scheduling will reduce the burden on participants and decrease the likelihood of loss to follow-up. Following completion of their involvement, participants will be remunerated with a CHF 40 grocery shopping voucher and receive their lung function, FeNO and blood pressure results.

### Observational Methods

The EPOCHAL study consists of 6 weeks of active data collection per participant, starting with an initial study nurse visit at the participant's home. During this 90-min visit, informed consent is given, and an intake questionnaire, including medical history and personal habits, is completed by participants. The first pulmonary (PFT, FeNO) and cardiac (BP, HRV) assessments will be conducted by the study nurse. The participant will then be scheduled for SPT at the Division of Allergy. This will involve a determination of sensitization to 17 pollen extracts (Table 1), although this number may be reduced in the event of pollen extract non-availability at the time of the appointment. Sensitization will be defined as a skin reaction ≥ 3 mm, in line with established European standards (85).

**Table 1:** Health outcomes collected in the EPOCHAL study (**bold**), assessments and questionnaires (***bold italics***), details on health outcomes (normal font).

**Collected health outcomes**	**Visit type and frequency**
**Medical history:** ***Intake questionnaire via electronic form (tablet)***	First nurse home visit (one time)
- Demographics: age, sex and gender - Socioeconomic status: education, employment - Medical history and medication use: allergies, asthma and other respiratory conditions, cardiac conditions, neurologic and psychiatric conditions, sleep conditions, COVID-19 - Previous allergy testing - Lifestyle: exercise, smoking, alcohol and caffeine consumption - Sleep habits	
**Pollen sensitization:** ***Skin prick test***	Visit to the Division of Allergy, University Hospital Basel (one time)
Sensitization to 17 different pollen types (alder, ash, beech, birch, cypress, grasses, hazel, sweet chestnut, mugwort, lichwort, oak, olive, plane, plantain, ragweed, sting nettle, and rye)	
**Pulmonary assessments**	Nurse home visits (6 times)
**Lung function:** ***Spirometry (EasyOne Air)***	
Expiration-only maneuver, 3 measurements separated by 2–3 min (best of 3 acceptable curves analyzed) - Forced expiratory volume in the 1^st^ second (FEV1) - Forced vital capacity (FVC) - Peak expiratory flow (PEF)	
**Airway inflammation:** ***Fractional exhaled nitric oxide test monitor (NO Breath)***	
Fractional exhaled Nitric Oxide (FeNO) in parts per billion, 3 measurements separated by 1–2 min (average of 3 measurements analyzed)	
**Cardiac assessments**	
**Heart rate variability:** ***1-lead electrocardiogram (ECG) on a chest belt (Actiheart 5)***	Nurse home visits (6 times)
Short term (8 min recording: first 5 min without motion artifacts analyzed), seated - Time domain: RMSSD (ms), PNN50 (%), SDNN (ms) - Frequency domain: High frequency (HF) and low frequency (LF) power, LF/HF ratio	
**Blood pressure:** ***Portable blood pressure monitor (Omron M3 Comfort)***	Nurse home visits (6 times) + 10-day participant measurements
Seated, 3 measurements separated by 1–2 min (average of 2^nd^ and 3^rd^ measurement analyzed) - Systolic BP (mmHg) - Diastolic BP (mmHg)	
**Physical activity and sleep:** ***Fitbit activity tracker (wristband)***	10-day participant measurements
- Calories burnt, kilometers, steps - Sleep duration, efficiency, and stages	
**Allergy symptoms**	Nurse home visits (6 times) + 10-day participant measurements
***Daily online questionnaire (about past 24 h) via URL link+Nurse home visit questionnaire via electronic form (tablet)***	
- Allergic symptom severity (nasal/ocular/pulmonary) - Sleep quality, mood, concentration, quality of life - Medication use - Caffeine, alcohol intake, smoking, exercise - Time spent outdoors	
**Cognitive performance:** ***Daily cognitive online games via URL link (Cambridge Brain Sciences)***	10-day participant measurements
1) Response inhibition/ability to focus (“Double Trouble” task score) 2) Verbal reasoning (“Grammatical Reasoning” task score) 3) Learning and memory (“Spatial Span” task score) 4) Attention (“Feature Match” task score) 5) Visuospatial processing (reaction times in tasks 1–4)	

*The second column shows when and how often these health outcomes will be collected*.

*RMSSD, root mean square of successive RR interval differences; PNN50, percentage of successive RR intervals that differ by more than 50 ms; SDNN, standard deviation of the inter-beat interval of normal sinus beats*.

*Device and software company details: Actiheart 5 (Camntech, Fenstanton, UK), Easy One Air (NDD Medizintechnik AG, Zurich, Switzerland), NO Breath (Bedfont Scientific Ltd, Harrietsham, UK), Omron M3 Comfort (OMRON Healthcare, Hoofddorp, Netherlands), Fitbit (Fitbit, Inc., San Francisco, US), Cambridge Brain Sciences (CBS, Toronto, Canada)*.

Each participant will have five subsequent 60-min home visits by a study nurse. Data collected will include: pulmonary (PFT and FeNO) and cardiac (BP and HRV) assessments as well as self-reported HRQoL, mood, and allergic symptom severity collected via an electronic questionnaire. These nurse visits will repeat at approximately the same time of day and will be spaced weekly, as it is assumed that participants will be naturally exposed to varying concentrations of ambient pollen.

During an overlapping 2-week period, participants will self-collect 10 days of data, to include: wear of a fitness/sleep tracker (periodic synchronization/transfer of data); participation in game-like assessments of cognitive performance; three consecutive measurements of BP using a device approved for home self-measurement; and completion of a brief online questionnaire regarding HRQoL and symptom severity. We estimate these items to collectively require 20 min of participant time per day.

The dates for the nurse home visits as well as the 10-day data collection period will be based on self-reported typical months of allergy symptomatology for individuals with IAR. For individuals without pollen allergy symptoms, data collection will occur during any 6-week block during the months of study eligibility (February through August), with an effort to have non-symptomatic participation spread approximately equally over the 7 months. Throughout the period of participant data collection (February–August 2021/2022), ambient pollen concentrations for the 17 chosen plant species will be furnished by the Federal Office of Meteorology and Climatology MeteoSwiss.

**Figure 1:**
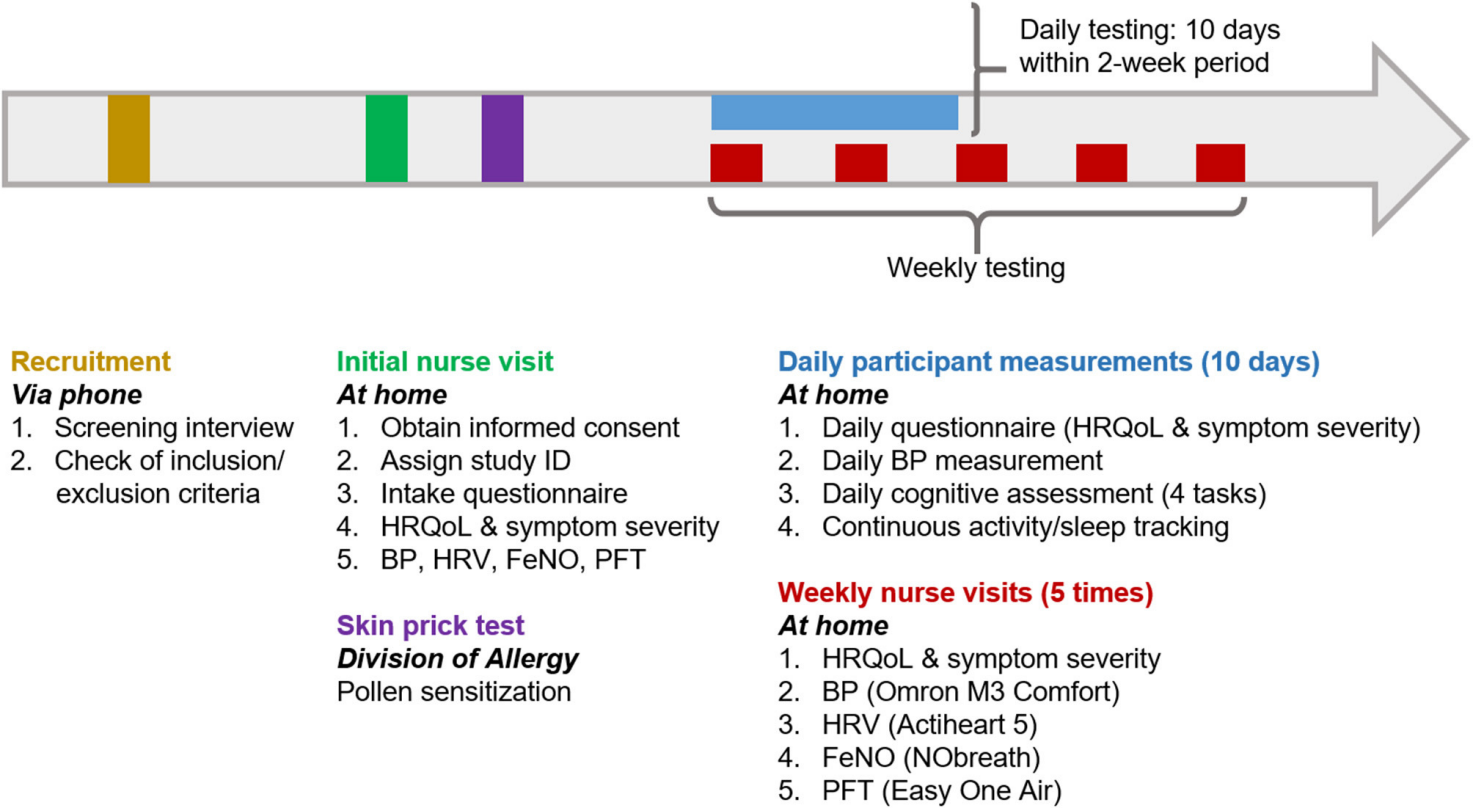
Visual representation of a typical EPOCHAL study participation timeline. This timeline will be initiated in February 2021. The skin prick test can be scheduled at any time after the initial nurse visit. The 10 days of participant data collection may occur within an overall 14-day period (this will allow for cessation of data collection over weekends, if preferred). This scheduling will be determined based on the participant's self-reported months of typical IAR symptoms (applicable to individuals with pollen sensitization) and acceptable months of enrollment (February through August) for all participants. HRQoL, Health-related quality of life; HRV, heart rate variability; BP, blood pressure; FeNO, fractional excretion of nitric oxide; PFT, pulmonary function test.

A typical participation timeline is presented in Figure 1. Details of all health assessments and questionnaires are listed in Table 1.

### Exposure Assessment

Pollen seasons differ in their temporality (see Supplementary Figure 1). Within Switzerland, daily pollen levels of 48 different plant species are measured at 14 stations operated by MeteoSwiss, the Federal Office of Meteorology and Climatology. For calendar years 2021 and 2022, we will obtain from MeteoSwiss the daily plant-specific pollen concentrations from the Basel station. The exposures of interest will be the same-day pollen exposure related to the 17 plants of interest, as well as the cumulative pollen burden (sum of daily concentrations of all 48 plants monitored by MeteoSwiss). We will additionally consider these same exposures on a 1- to 7-day lag period.

It is understood that the published pollen data from the MeteoSwiss Basel monitoring station will not precisely match the unique pollen exposure of any particular participant, which can be locally influenced by green space, topography, altitude, and local plant density and variety. The EPOCHAL study will take into account the temporal change in pollen concentration over an approximate 6-week period for each participant, which will allow for enough pollen concentration variability to understand any potential pollen dose-response relationships. The EPOCHAL study will evaluate the confounding properties of both air pollution and weather on the six health domains (sections Pulmonary Outcomes, Cardiovascular Outcomes, Cognitive Performance, Sleep, Health-Related Quality of Life, and Allergic Rhinitis Symptom Severity). This goes well beyond what prior epidemiologic research investigated.

### Data Analysis

The planned analysis will study the effect of MeteoSwiss pollen concentrations on the six major health outcomes (pulmonary, cardiac, cognitive, sleep, HRQoL, and symptom severity). We will analyze how these outcomes vary between pollen-sensitized and non-sensitized individuals as well as how these outcomes vary in IAR individuals at varying levels of pollen exposure. For all primary outcomes, the null hypotheses will be that:

1. There is no difference in outcome X between individuals with or without pollen sensitization(s).
2. There is no difference in outcome X for pollen-sensitized individuals at varying levels of pollen exposure.

The alternative hypotheses will be that there are significant differences for outcome X. The statistical significance level will be set as two-sided α = 0.05. Analyses will be conducted using the lme4 R package for mixed models, considering that repeated measurements will be available for the same individual under different conditions.

Pulmonary, cardiac, cognitive, sleep, HRQoL, and symptom severity outcomes will be analyzed as a function of same-day pollen exposure as well as multi-day lag (up to 7 days). In addition, we will correct for the following potential confounders:

- Specifically for the cognitive testing, a potential learning effect that repetition in cognitive testing can have on individual participants' scores.
- For cognitive and HRQoL outcomes, the previous night's sleep quality.
- Class(es) of allergy medication used by participants in the preceding 24-h period.
- Effect of different outdoor exposure periods (example: <1 h, 1–4 h, >4 h) on health outcomes.
- Environmental confounders such as weather conditions and air pollution.

We will use a generalized linear mixed effects regression model with a random intercept to account for individual baseline differences in health outcomes. If a dose-response effect is found between pollen exposure and any health outcome, we will investigate whether there is a pollen concentration above which no further health outcome effect is noted or a pollen threshold below which there is no indication of such an effect. We will analyze potential differences in the primary outcomes related to plant pollen mono- vs. polysensitization. Further, we will look at effect modifiers to ascertain if dose-response relationships are different between age and socioeconomic status strata, for men and women, for smokers vs. non-smokers, and for asthmatics vs. non-asthmatics.

#### Sample Size Calculation

To perform the sample size calculation, the power two means command was used in Stata with the inputs listed in Table 2, a significance level of 0.05, and power of 0.80. A ratio of pollen allergic/non-allergic population (kratio) of 3:1 was set.

**Table 2:** Sample size calculations for cardiovascular, pulmonary and cognitive outcomes.

	**Mean 1 (Std dev)**	**Mean 2 (Std dev)**	**M**	**rho**	**Sample sizes for main effect of pollen on outcome**		
	**Allergic**	**Non-allergic**			***N* allergic**	***N* non-allergic**	***N* total**
**Cardiovascular outcomes**							
Systolic BP (mmHg) (30)	129.9 (13.2)	123.6 (13.3)	18	0.8	114	38	152
RMSSD (ms) (45)	48 (27)	31 (20)	6	0.8	44	15	59
PNN50 (%) (45)	29 (8)	23 (9)	6	0.8	56	19	75
SDNN (ms)^a^	1 (0.09)	1.04 (0.09)	6	0.7	720	240	960
HF (43)	5.9 (1.1)	5.3 (1.0)	6	0.6	62	21	83
LF (43)	6.2 (1)	6.4 (0.8)	6	0.4	287	96	383
LF/HF (43)	0.3 (0.7)	1.1 (0.7)	6	0.6	17	6	23
**Pulmonary outcomes**							
FEV_1_ (ml)^a^	1 (0.07)	1.07 (0.06)	6	0.8	150	54	204
FVC (ml)^a^	1 (0.07)	1.07 (0.06)	6	0.8	150	54	204
FEV_1_/FVC (%)^a^	1 (0.07)	1.07 (0.06)	6	0.8	150	54	204
FeNO (ppb) (29)	22.5 (10)^b^	17.0 (10^a^)	6	0.8	87	29	116
**Cognitive outcomes**							
Double trouble (reaction time, ms)^c^	2,437 (757)	2,031 (757)	14	0.7	79	27	106
Grammatical reasoning (score)^d^	17.0 (5.2)	16.1 (5.2)	14	0.6	660	220	880

*Mean 1 (and standard deviation) is presented for the allergic population. Mean 2 (and standard deviation) is presented for the non-allergic population. M, data points per individual; rho, intraclass correlation coefficient. RMSSD, PNN50, and SDNN represent time domain measures of HRV; HF and LF represent power domain measures of HRV; LF/HF is a calculated ratio*.

a *Estimated by EPOCHAL team*.

b *No standard deviation given in publication, EPOCHAL team estimation presented*.

c *20% increase in reaction time compared to non-allergic group estimated from Trikojat et al. (53)*.

d *5.5% decrease in verbal memory compared to non-allergic group, estimated from Trikojat et al. (53)*.

For sample size estimation for cardiovascular outcomes, a rather conservative high estimate for the intra-class correlation coefficient (rho) on the individual level was used. It was derived from a dataset that included three repeated HRV and BP assessments within the same individual (6 months apart) (86). Rho and mean for non-allergic population of the cognitive outcomes was assessed by analyzing the correlation between 10 day-to-day measurements from two cognitive tests found in a Swiss TPH study dataset. Rho for pulmonary outcomes was conservatively set to 0.8 (no reference dataset available).

Two group means (allergic vs. non-allergic population) are compared since this is the distinction made in the available literature. The group means are used to approximate the values of a high-pollen day when an allergic person would be symptomatic vs. a low to no-pollen day when the same person is not symptomatic.

As noted in Table 2, the sample size calculations range from 23 to 960 participants to adequately power the pulmonary, cardiac, and cognitive outcomes. We have chosen to target a sample size of 400 adults as it will adequately power the majority of our endpoints within these health domains and will compensate for participant drop-out.

## Ethics and Dissemination

Ethical clearance has been obtained from the Ethics Committee for North-Western and Central Switzerland (EKNZ number 2021-00151). This study is a research project involving human subjects with the exception of clinical trials and falls under the Risk Category A in the Human Research Ordinance of the Human Research Act (87). Informed consent will be obtained from all participants prior to entering the study, compliant with the Declaration of Helsinki (88). Participant information and informed consent forms will be available in German and English.

### Data Management

#### Data Protection

Data collected in this panel study is subject to compliance with the Federal Data Protection Act (DSG) as well as the EU General Data Protection Regulation (GDPR). All personal data will be coded by assigning a unique study ID to each participant. This study ID will be used for all digitally collected questionnaire data as well as data input into EasyOne and Actiheart software for spirometry and HRV assessment, respectively. The data gathered by Cambridge Brain Sciences (CBS), the software provider of the daily cognitive games, and the Fitbit wristband tracker are stored on the device as well as servers belonging to these companies. Platform-specific IDs will be generated for each participant of the study, which the companies cannot trace to the individual person or their coded study ID. Through these platform-specific IDs, the companies will be aware that a single participant made repeated measurements (of e.g., sleep or cognitive function), but they will not be able to trace who this person is. All data is saved on a secure internal server that can only be accessed by study team members and will be stored for 10 years.

#### Cambridge Brain Sciences and Fitbit Data

We will take special care to limit data sharing and data use to the minimum necessary information needed to conduct the study. In case of CBS, the company will not obtain any data other than the CBS-specific ID and the test results obtained through the platform itself. In the case of Fitbit, besides a Fitbit-specific ID, the device requires input of sex, height, weight and date of birth in order to function. Sex, height, and weight cannot be used to uniquely identify people, and we will use a default date of birth as 1 July of the actual year of birth to minimize any possibility of identification through this data point. The study nurse will inform participants about these specific data safety measures taken during the informed consent process.

It is possible that coded data collected by CBS and Fitbit is used for other purposes (see privacy policies of Fitbit: https://www.fitbit.com/global/us/legal/privacy-policy#how-info-is-shared and CBS: https://www.cambridgebrainsciences.com/privacy-policy). We will make all participants aware of the implications of sharing data with the aforementioned companies. It will be explicitly discussed that CBS and Fitbit have their own data privacy policies, which are beyond the control and responsibility of Swiss TPH and EPOCHAL study staff. Participants will be directed to read these policies should they have questions about the hosting of their personal data, including whether the data may be potentially hosted abroad.

### Ethical Considerations and Risks

Participants will benefit from this study through knowledge of their personal pollen sensitization profile. They also gain insight into their cardiovascular and pulmonary health and sleep habits. We will provide each participant with a summary document of their personal PFT, FeNO, and BP outcomes within 3 months of their study participation completion. They may be altruistically motivated by the knowledge that EPOCHAL study results could lead to environmental and health policy improvements within Switzerland and globally. They will receive a symbolic monetary compensation of CHF 40 for taking part in the study.

Premature dropout because of repeated testing over several weeks is a risk, which we mitigate by minimally invasive tests and conducting the vast majority of health assessments conveniently at the participants' homes. Furthermore, personal contact and clear communication during recruitment should ensure engaged participation.

There is little potential risk for the study participants. All pulmonary and cardiac measurements are minimally invasive and considered low-risk, and utilization of the study devices has no potential to change pulmonary or cardiac physiology. There is an extremely low risk of a systemic reaction to the SPT, which can be reversed with epinephrine injection, which will be available at the Division of Allergy testing location. The presence of a medical doctor in the building is required and will be assured.

If a participant repeatedly shows abnormally high BP according to the definitions of hypertension set-forth by the European Society of Cardiology Guidelines (89), or abnormally reduced lung function according to the European Respiratory Society (90), they will be advised to seek medical advice. If the participant is already under treatment (e.g., blood pressure lowering or asthma medication) and the pulmonary/cardiac assessments are repeatedly abnormal, we will advise the participant to consult with their treating provider. For participants with pollen sensitization who desire medical management for allergy symptoms, we will provide contact information of local allergists, while making explicit that such treatment cannot be offered as part of participation in this study.

To mitigate infection risk with COVID-19, a viral disease transmitted by droplets and aerosols, we will implement protective measures, such as the wearing of gowns, masks, gloves, and goggles by the study nurses, keeping a 1.5-meter distance whenever possible, and thoroughly disinfecting equipment after each use.

## Discussion

EPOCHAL is a novel, single site, observational study which seeks to associate daily pollen exposure with a diverse range of health outcomes. Given that up to 20% of the Swiss population is affected by pollen-triggered AR, any significant difference in pulmonary, cardiovascular, cognitive, sleep, HRQoL, or symptom severity outcomes is of high public health importance. Longer pollen seasons, larger cumulative pollen concentrations, and invasion of more allergenic and non-native species increase the future probability of higher population prevalence of pollen allergy. While previous research has suggested a relationship between IAR and systemic health effects, little is known about how these health outcomes are modulated by variable concentrations of pollen or the number of plant pollen sensitizations on SPT. It is also not understood whether adults *without* pollen sensitization manifest changes in pulmonary, cardiovascular, cognitive, sleep, and HRQoL domains in response to low, moderate, or high pollen exposure. EPOCHAL aims to close these knowledge gaps through a well-powered, longitudinal study during the 2021 and 2022 Basel pollen seasons.

There are enormous direct and indirect health costs associated with each of the aforementioned health outcomes, and quantitatively measuring pollen-triggered hazards to population health is the fundamental driver behind this project. This project aims to understand *if* and the *extent to which* a higher pollen concentration is associated with differences in BP, HRV, pulmonary function, lung inflammation, cognitive function and processing speed, sleep quantity and quality, HRQoL, and AR symptom severity. Based on prior literature, we hypothesize that increasing pollen concentration will be associated with decreases in pulmonary function, cognitive function, processing speed, sleep duration and efficiency, and HRQoL. Conversely, we expect that increasing pollen concentration will be linked with increases in airway inflammation (FeNO) and self-reported symptom severity. The relationship between HRV as well as BP, AR, and pollen exposure is not well-defined and clarifying this association is a particularly important aspect of EPOCHAL. Furthermore, understanding whether these pollen-related changes are also manifested in a non-sensitized adult population makes the EPOCHAL project highly relevant for the general population and public health system.

Inflammation related to AR extends beyond the upper respiratory tract (91, 92). Systemic circulation of cytokines and activated immune cells, such as T lymphocytes, has been used to explain the lower respiratory and neurologic (cognitive and HRQoL) manifestations of AR (92). Beyond these systems, cutaneous reactivity to pollen—readily demonstrated through SPT—shows that the skin is also affected through systemic processes (91). Whether cardiac tissue is likewise affected by systemic inflammation following pollen exposure is not yet established.

Whereas previous studies have been limited by cross-sectional or two time point designs, the EPOCHAL study will collect pollen and health data at 16 time points within a period of relevant and abundant pollen exposure in Basel, Switzerland (February through August). The serial measurements uniquely proposed by EPOCHAL will allow for outcome investigation along a continuum of differential pollen exposure. Statistical analysis will specifically consider sensitive subgroups which may show variable results, for example: younger vs. older age strata; asthmatics vs. non-asthmatics; and pollen mono- vs. polysensitized.

A strength of our design is the consideration of same-day as well as lag (1–7 day) pollen exposure. This feature takes into account that some systemic inflammatory effects will not manifest immediately. Another strength is the inclusion of up to 17 relevant, allergenic pollens. This will allow for discovery of dose-response relationships, plateaus, and thresholds between pollen concentration and health parameters that may be limited to specific plants. The EPOCHAL study will also consider important confounders, which have been minimally explored in previous research, such as: air pollution exposure, weather, sleep quality and quantity from preceding night (for cognitive and HRQoL outcomes), caffeine intake, and use of allergy medications (sedating, excitatory, and disease-modifying).

One limitation of previous studies which we overcome is objective confirmation of pollen sensitization through SPT rather than reliance on self-report. In conjunction with self-reported symptom severity data, we can determine how the SPT profile is associated with the intensity or significance of systemic health effects. If such a relationship exists, this has the potential to greatly impact IAR medical management at the time of pollen allergy diagnosis.

Another strength of the EPOCHAL study is the minimization of exclusion parameters in order to best approximate “real world” conditions. Whereas, some prior studies have disallowed allergy medication use and excluded asthmatics and individuals with hypertension, we believe our more inclusive approach to participant enrollment will generate results that are more relevant and applicable. One interesting feature of the EPOCHAL design is the targeted inclusion of ~25% non-allergic adults. Our rationale extends beyond the need for data comparison between sensitized and non-sensitized participants. To our knowledge, there is a paucity of research on systemic health effects of pollen exposure on non-sensitized adults. Given the global trends toward longer and more intense pollen seasons, this knowledge is important for the medical and public health communities.

The EPOCHAL project aims to inform public health policies, particularly those which mitigate risk for the most vulnerable groups; shape environmental policies aimed at minimizing exposure to particularly allergenic plant species; and decrease the economic burden of IAR. Furthermore, with this work, we will make a significant step forward in providing personalized prevention recommendations that could greatly improve the quality of life of the pollen-allergic population. The health outcome information is crucially needed for accurate timing of population health alerts. The EPOCHAL study, while focused on an adult population in the Basel, Switzerland region, is widely generalizable to the wider European and global communities.

## Ethics Statement

The EPOCHAL study involves human participants and was reviewed and approved by the Ethics Committee for North-Western and Central Switzerland (EKNZ number 2021-00151). The participants will provide their written informed consent prior to participation in this study.

## Author Contributions

AB, SG, KH, and ME contributed to conception and design of the study. AB wrote the first draft of the manuscript and created the figures and tables. AB and SG wrote sections of the manuscript. All authors read and approved the submitted version.

## Conflict of Interest

The authors declare that the research was conducted in the absence of any commercial or financial relationships that could be construed as a potential conflict of interest.

## Abbreviations

BP: Blood Pressure
CBS: Cambridge Brain Sciences
EPOCHAL: Effects of Pollen on Cardiorespiratory Health and Allergies
FeNO: Fractional excretion of Nitric Oxide
HRQoL: Health-related Quality of Life
HRV: Heart Rate Variability
(I)AR: (Intermittent) Allergic Rhinitis
PFT: Pulmonary Function Test
SPT: Skin Prick Test.
