# Supplementary material for: Rationale and Design of a Panel Study Investigating Six Health Effects of Airborne Pollen: The EPOCHAL Study
Source: Front Public Health. 2021 Jun 18;9:689248. doi: 10.3389/fpubh.2021.689248 (PMC8249754; doi:10.3389/fpubh.2021.689248)
Supplement: Supplementary file 1 [file Data_Sheet_1.PDF]

Supplementary Material

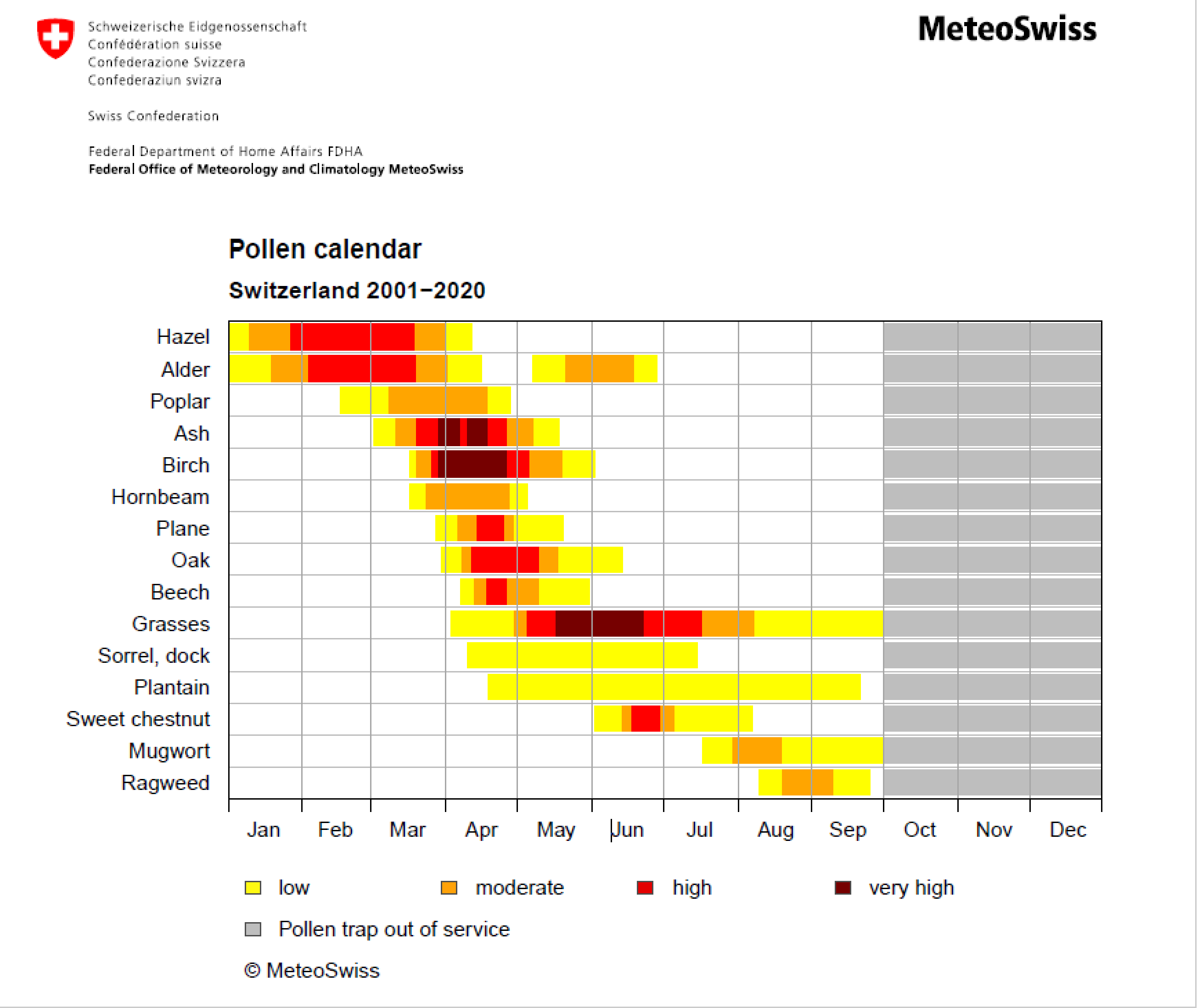

Supplementary Figure 1. Average pollen seasons (years 2001-2020) for 15 allergenic pollen species occurring in Switzerland. Source: MeteoSwiss

Meteoswiss *Pollen calendar Switzerland 2001-2020* [Online]. Available: https://www.meteoswiss.admin.ch/home/climate/the-climate-of-switzerland/pollen-information.html [Accessed 10 May 2021].
